# Supplementary material for: Simple and robust 3D MINFLUX excitation with a variable phase plate
Source: Light Sci Appl. 2024 Jun 7;13:134. doi: 10.1038/s41377-024-01487-1 (PMC11161593; doi:10.1038/s41377-024-01487-1)
Supplement: Supplementary file 1 — Supplemental material [file 41377_2024_1487_MOESM1_ESM.pdf]

Simple and robust 3D MINFLUX excitation with a variable phase plate

Takahiro Deguchi[1], Jonas Ries[1,2,3,4,*]

[1] European Molecular Biology Laboratory, Cell Biology and Biophysics, Heidelberg, Germany.
[2] Max Perutz Labs, Vienna Biocenter Campus (VBC), Vienna, Austria.
[3] University of Vienna, Center for Molecular Biology, Department of Structural and Computational Biology, Vienna, Austria.
[4] University of Vienna, Faculty of Physics, Vienna, Austria.
* Correspondence: Jonas.ries@univie.ac.at

Supplementary Figures

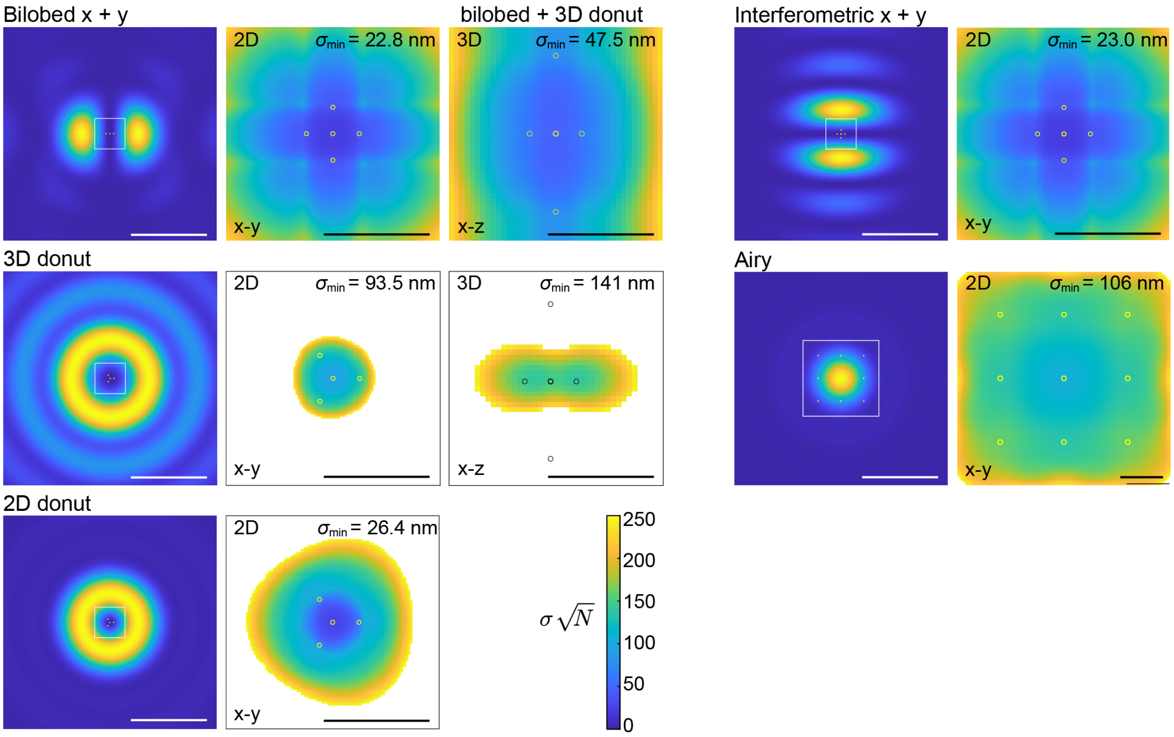

Supplementary Figure 1. Theoretical localization precision limit for different PSFs. *Cramér-Rao bound (CRB) calculations for 2D MINFLUX and 3D MINFLUX with commonly used PSFs, extending code provided by Masullo et al.[17] (see* ***Methods, Data and code availability).*** *The calculated localization precisions are normalized by* $\sqrt{N}$*, and thus report the localization precision for a single photon. Circles indicate the positions of the minimum during probing. Simulation parameters: scan range L = 50 nm in the lateral and* L_z *= 150 nm in the axial direction, except for the Airy PSF with L = 300 nm for a 3 x 3 scan pattern. Offset (value at PSF minimum) 0.5% of the maximum intensity of the Airy PSF. Scale bars 500 nm (PSFs), 100 nm (CRB maps).*

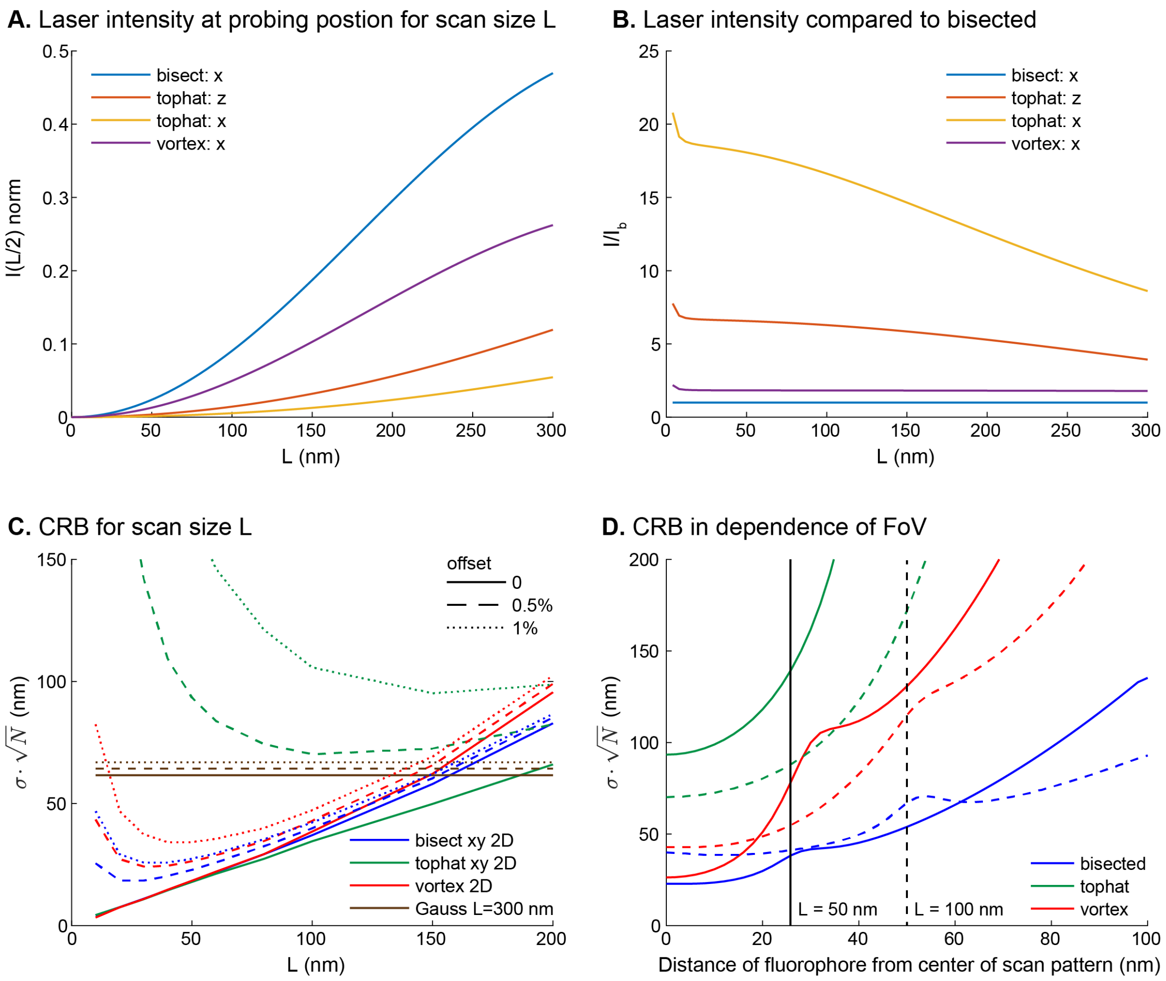

Supplementary Figure 2. Dependence of localization precision on scan range and distance of the fluorophore from the center of the scan pattern. *CRB calculations are described in the* ***Methods*** *section.* ***A:*** *Intensity at the probing position for scan sizes L,* ***B:*** *Intensity at probing position normalized by intensity for the bisected phase pattern (bilobed PSF), indicating that especially the 3D donut, but also the vortex PSF require higher laser powers for a similar signal. This leads to an increased offset due to an imperfect PSF, higher auto-fluorescence and out-of-focus fluorescence and consequently to an increased intensity at the minimum.* ***C:*** *Maximum localization precision (CRB, normalized by photons) in dependence on scan size L and offset for different MINFLUX PSFs, indicating that PSFs with a larger size are more sensitive to background.* ***D:*** *Maximum lateral 2D localization precision in dependence on the distance of the fluorophore to the center of the scan pattern, i.e., the field of view for a scan range of L = 50 nm (solid line) and L = 100 nm (dashed line). The vertical lines denote the maximum position of the intensity minimum. In contrast to donut PSFs, the bisected PSFs allow for localizing fluorophores even outside the scan range with an acceptable precision. Thus, the number of iterations with decreasing L can be reduced, which leads to improved speeds and less photons lost during the initial coarse localization process. Background offset 0.5% of the maximum intensity of the Airy PSF.*

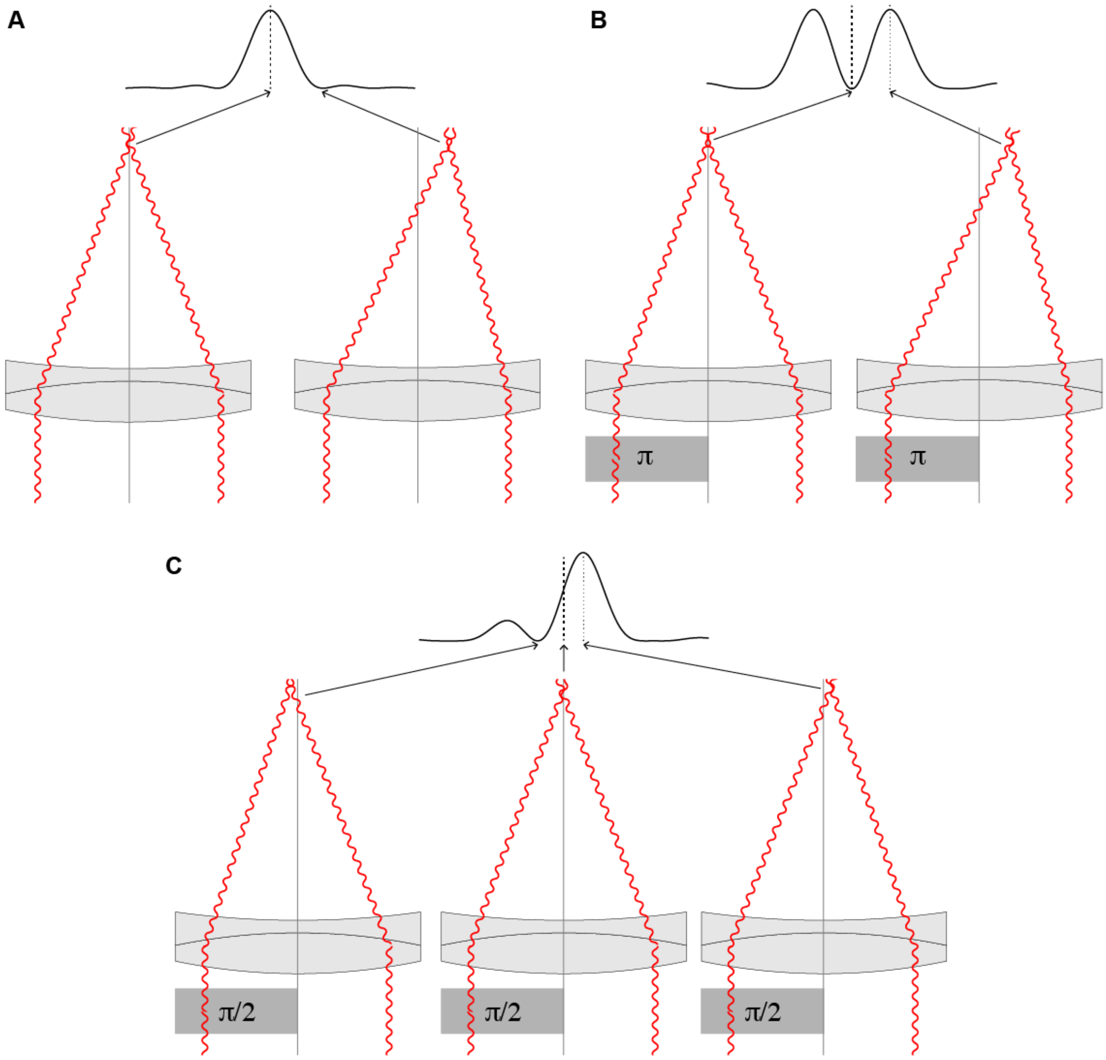

Supplementary Figure 3. Illustration of how PSFs with a local minimum are generated. A: *In the focus of a lens on the optical axis, all beams have the same path lengths and thus interfere constructively to result in an intensity maximum. An off-axis point has a longer path length on one side and a shorter one on the other side, leading to destructive interference and an intensity minimum.* ***B:*** *A phase shift for half of the beams leads to destructive interference in the focus, and symmetric off-axis with constructive interference, i.e., intensity maxima.* ***C:*** *A general phase shift different from* **π** *leads to points of constructive and destructive interference at lateral positions that can be chosen by the phase shift.*

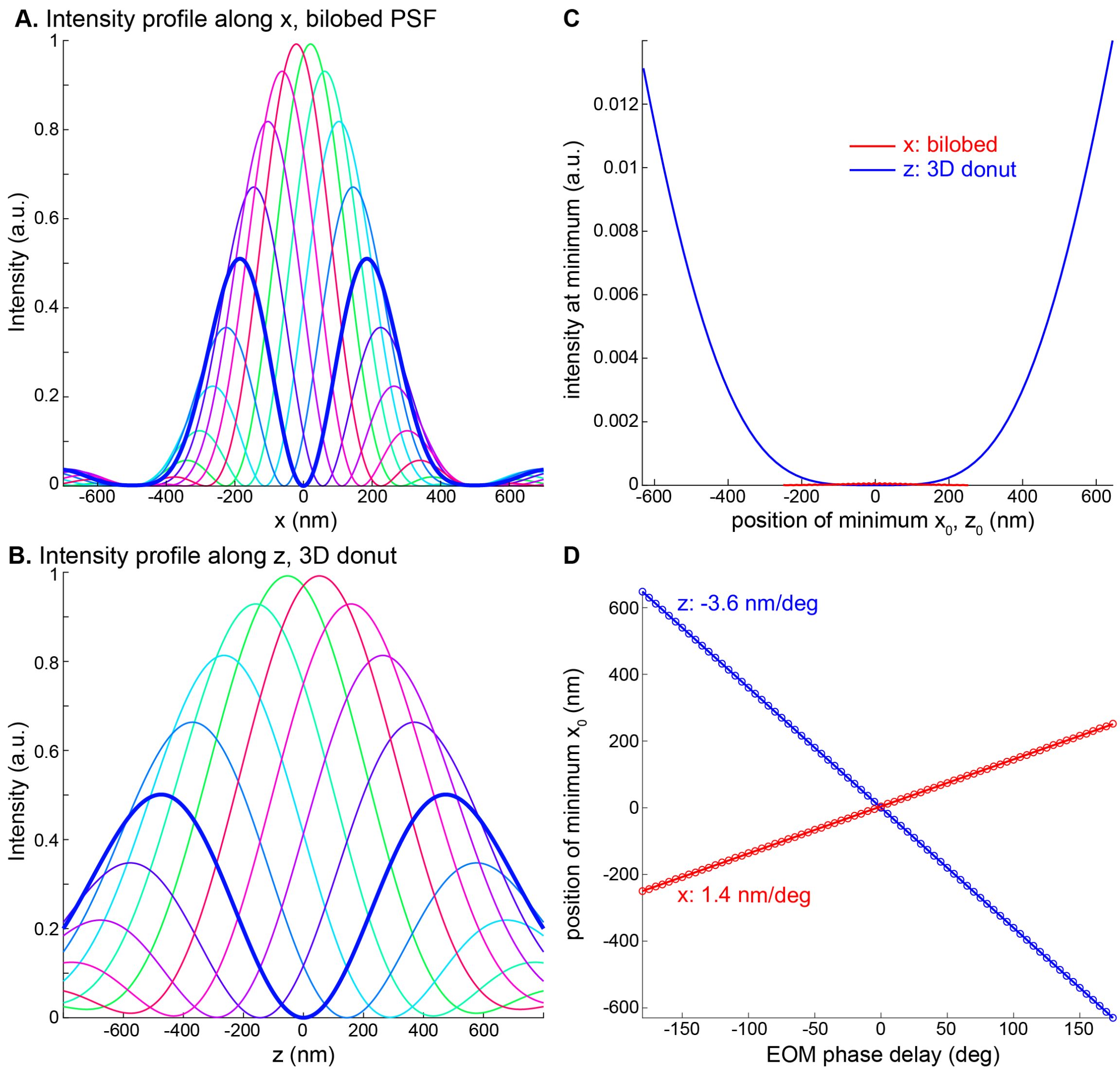

***Supplementary Figure 4: Intensity profiles through simulated MINFLUX PSFs** for an NA 1.35 system. PSFs were calculated using full vectorial wave propagation as described in **Methods**, showing that the performance of the excitation module remains high in a high-NA microscope implementation. **A: bilobed PSF** from a bisected phase pattern for different EOM phases with a spacing of 40°. **B: 3D donut PSF** from a tophat phase pattern used for z localization for different EOM phases with a spacing of 40°. **C: Intensity at minimum** in dependence of the position of the minimum for the bilobed PSF and the 3D donut, respectively. The intensity minimum for the x and y PSFs remains zero within numerical precision, independent of the scan range. **D:** Position of the minimum vs EOM phase delay. With a half-wave voltage of 210 V for the EOM, this corresponds to 1.2 nm/V (x,y) and -3.1 nm/V (z).*

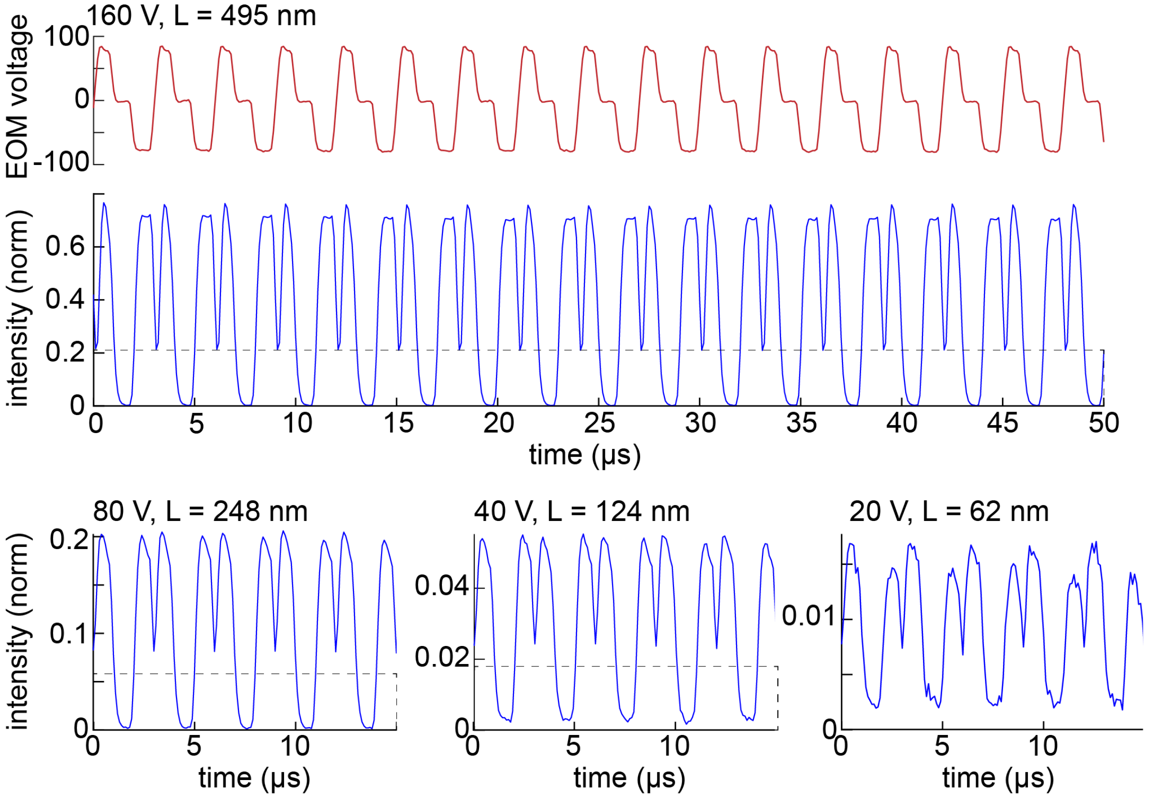

***Supplementary Figure 5: Intensity measurements with a pinhole and photo diode, 3D donut and z scanning**, see Figure 3**.** Intensity (normalized to the intensity measured for a flat phase pattern) recorded through the pinhole for three different voltages, each lasting for 1 µs, the minimum of the phase pattern is positioned at three distinct positions around the fluorophore. L is the diameter of the scan pattern when implemented in a microscope. By changing the amplitude of the voltage, the scan range L can be reduced to increase the localization precision. The dashed line indicates the zoom region for the subsequent panel.*

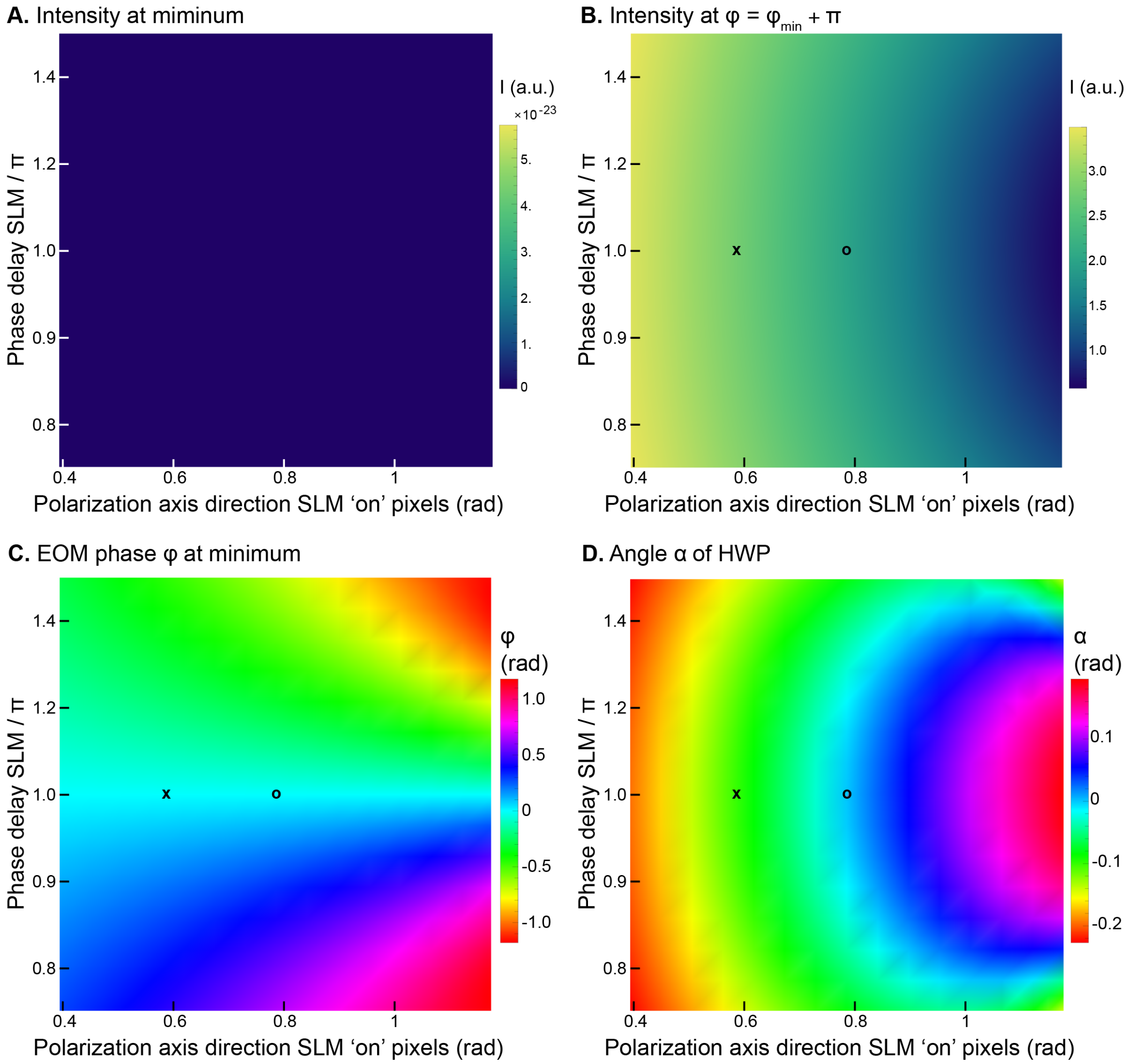

Supplementary Figure 6. Compensation of SLM imperfections. *By inserting an additional halfwaveplate (HWP) in the beam path, we can entirely compensate for an imperfect phase delay* $\boldsymbol{\phi_{SLM}}$ *or polarization axis* $\boldsymbol{\alpha_{SLM}}$ *of the SLM. see* ***Methods*** *and* ***Data and code availability*** *for the details on the modelling.* ***A:*** *We numerically optimized the rotation angle* $\boldsymbol{\alpha}$ *of the HWP and the EOM phase* $\boldsymbol{\varphi}$ *to minimize the intensity in the PSF center and found that for a large range of values the center intensity was zero within the calculation precision.* ***B:*** *Intensity for the optimal* $\boldsymbol{\alpha}$ *for an EOM phase of* $\boldsymbol{\varphi + \pi}$ *as a measure for the efficiency (light transmission) of the setup.* ***C:*** *EOM phase* $\boldsymbol{\varphi}$ *and* ***D:*** *angle of the HWP* $\boldsymbol{\alpha}$ *for a given combination of SLM phase delay and polarization axis. 'o' indicates parameters for an optimal SLM, 'x' indicates the parameters in our experiment.*

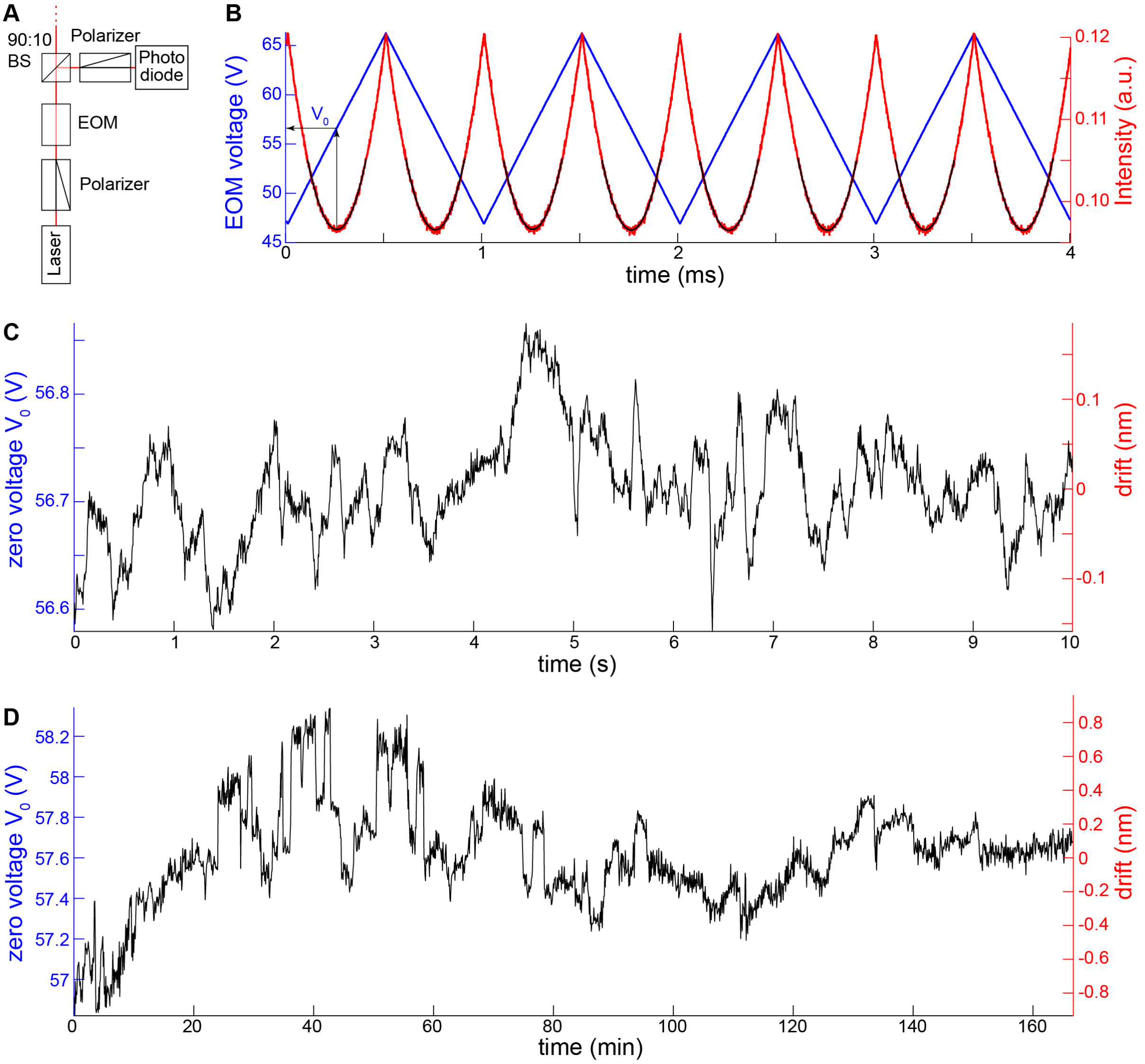

Supplementary Figure 7: Stability of the EOM phase. A: Setup. *10% of the beam is split off with a non-polarizing beam splitter (BS), passes a Glen-Thompson polarizer and is detected with a photo diode.* ***B: Measurement of the zero voltage V_0.*** *The EOM voltage is scanned in a triangle pattern around* V_0 *(blue line). A quadratic fit (black line) of the intensity (red line) is used to determine the position of the intensity minimum, which occurs at* V_0. ***C: Drift over seconds.*** V_0 *and the corresponding drift as calculated for a NA 1.35 objective over time.* ***D: Drift over hours.*** *Same as* ***C****, but for a measurement time of 167 minutes.*

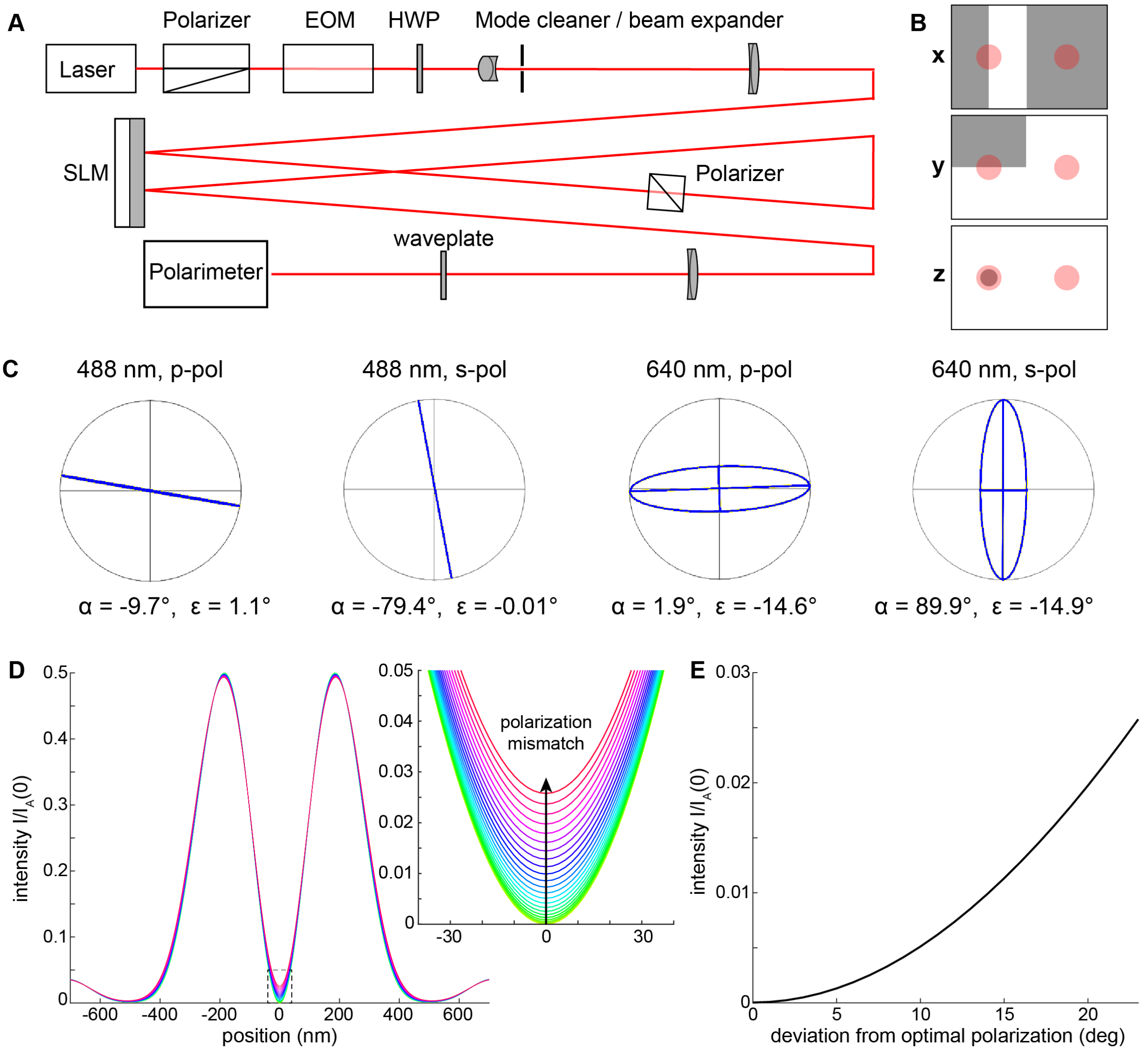

Supplementary Figure 8: Polarization matching by second reflection off the SLM. *Our bilobed PSFs require the polarization axis to be parallel to the phase boundary of the SLM, otherwise the intensity minimum increases, leading to a reduced localization precision.* ***A: Double-bounce beam path*** *to rotate the polarization of the outgoing beam.* ***B: Phase patterns on the SLM*** *for the x, y and z PSFs. The shaded area are the 'on'-pixels. The red circles indicate the first and second reflection of the laser beam.* ***C: Measured polarization state*** *after a waveplate inserted to distribute the polarization error between the two polarization components.* ***D: Simulated profiles*** *for the bilobed PSF for an increasing polarization mismatch (from 0° to 23° with 1° steps between profiles).* ***E: Contrast of the PSF minimum in dependence on polarization mismatch****. The intensity is normalized by the maximum intensity of a beam created with a flat phase mask and equal intensity.*
